# Surveillance Bias in Child Maltreatment: A Tempest in a Teapot

**DOI:** 10.3390/ijerph14090971

**Published:** 2017-08-28

**Authors:** Brett Drake, Melissa Jonson-Reid, Hyunil Kim

**Affiliations:** Brown School of Social Work and Public Health, Washington University in St. Louis, Campus Box 1196, Washington University in St. Louis, One Brookings Drive, Saint Louis, MO 63130, USA; jonsonrd@wustl.edu (M.J.-R.); hyunilkim@wustl.edu (H.K.)

**Keywords:** surveillance bias, child abuse and neglect, child maltreatment, nurse home visiting, visibility bias

## Abstract

*Background:* Children are believed to be more likely to be reported for maltreatment while they are working with mental health or social service professionals. This “surveillance bias” has been claimed to inflate reporting by fifty percent or more, and has been used to explain why interventions such as home visiting fail to reduce official maltreatment reporting rates. *Methods:* We use national child abuse reporting data (*n* = 825,763), supplemented by more detailed regional data from a multi-agency administrative data study (*n* = 7185). We determine the percentage of all re-reports made uniquely by mental health and social service providers within and across generations, the report sources which could be subject to surveillance bias. *Results:* At three years after the initial Child protective services (CPS) report, the total percentage of national reports uniquely made by mental health or social service providers is less than 10%, making it impossible that surveillance bias could massively inflate CPS reporting in this sample. Analysis of national data find evidence of a very small (+4.54%) initial surveillance bias “bump” among served cases which decays to +1.84% within three years. Our analysis of regional data showed similar or weaker effects. *Conclusions*: Surveillance bias effects appear to exist, but are very small.

## 1. Introduction

Surveillance bias (SB), sometimes termed “detection bias” or “visibility bias”, is an important but understudied construct in child maltreatment research. It is the idea that children who receive services will have artificially and substantially increased rates of official Child Protective Services (CPS) reports due to their increased visibility to professional reporters, specifically mental health and social service providers. At least three kinds of SB have been claimed to exist. First, children who are being served by a program like nurse home visiting, may be more likely to be reported simply because they are being “directly” surveilled by the home visitor [1,2,3,4,5,6] or “indirectly” surveilled [4,5] by a secondary service provider to whom the child is referred to by the initial provider. Finally, children contacted by CPS are often believed to have a high risk of ongoing or future surveillance. This latter type of bias has been asserted to be intergenerational in nature. When children who are reported grow up and have their own children, it has been suggested they may be at increased risk of being reported as perpetrators simply because they had prior child welfare system contact [7].

This seemingly arcane issue is of critical practical and public policy importance [8], as “surveillance bias is often invoked to explain null or paradoxical findings in child abuse prevention or intervention trials.” [5]. SB is often cited in the home visiting and early head start literature as an explanation for why treated families do better on various study-generated measures but generally do not show lower rates of official maltreatment reports [2,4,9]. Howard and Brooks-Gunn state “the difference in surveillance between the treatment and control groups probably explains why so few home-visiting programs have measurable effects on rates of abuse and neglect” [10]. A recent study extended this assertion intergenerationally, finding “implications for child protective service systems that may be disproportionately scrutinizing families with past histories of child maltreatment” [7].

In any discussion of SB it is essential to recognize that SB cannot exist if the reported child is additionally reported by a source not subject to SB, such as a neighbor. This is because such “non-unique” children are reported anyway and the potential SB is redundant. No new child is identified [5].

### 1.1. Work Cited in Support of Surveillance Bias

In one of the more heavily cited works in the SB literature, Bilukha and colleagues [2] conclude that SB could be an explanation for null intervention effects. This assertion was based on just two small published intervention studies, neither of which claimed statistically significant evidence of an important SB effect [11,12]. Dawson and colleagues [12] examined a home visitation program for low income women and found that 5 children out of 67 intervention cases compared to 1 in 44 of the controls were reported for maltreatment over 12 months, a non-significant difference (Fisher’s Exact Test *p* = 0.4). In a post-hoc assessment the authors found that three of the five treatment reports were generated directly by the home visitor’s concerns, an example of direct surveillance. This was seen as resulting in a SB effect of +150% (Five total reports minus 3 SB reports equals = two “non-SB” reports, and relative to two non-SB cases, three SB cases provide a 150% increase).

Brayden and colleagues [11] tested another home visitation intervention with mothers at high risk of maltreatment. This study did not include all reports to child protective services, only reports subsequently judged to be valid by independent physician raters. According to this method 9.2% of the treatment group (*n* = 141) and 6.6% of the controls (*n* = 122) were reported as physically abused, a nonsignificant difference. 10.6% of treatment families and 4.1% of the controls were neglected; this was reported as significant despite the confidence interval spanning one (0.98–7.91). Brayden and colleagues also examined the source of reports and found that 30% of physical abuse and 15% of neglect reports for intervention cases were made by program staff. They adjusted for this possible SB effect and still found no significant program effect. Finally, they found that program clients had twice as many health care visits as control clients after the child was born, highlighting the possibility of indirect surveillance.

Subsequent to their review of the above studies, Bilukha et al. [2] assert that Dawson and colleagues [12] found a SB effect of +150% and Brayden et al. [11] found evidence of a +80% SB bias. The review authors then suggest what they call a “conservative” “abuse/neglect adjusted” rate (+50%) that researchers could apply to non-served comparison groups in the future to adjust for SB. The means of translating the 150% and 80% estimates to a 50% adjustment was unclear, but even the higher rates would likely not have altered the null statistical findings from the original studies. This may explain why later studies that checked for a SB effect in controlled trials of home visitation were unable to find a meaningful impact [6,13]. While MacMillan [13] did observe that reports made on two of their treatment group families could be attributed to SB, excluding these two families from the treatment group did not change the overall null finding for the intervention. Nor did MacMillan find discrepancies between their internal measures of risk and CPS outcomes as mentioned in other studies [4,9].

While SB is traditionally discussed relative to intervention studies, Widom and colleagues [7] extended this as a possible rationale for anomalous findings between official and self-reported maltreatment in a longitudinal study. Compared to matched controls, adult subjects (G1: 1st generation) with childhood histories of court adjudicated maltreatment were more likely (Adjusted Odds Ratio (AOR) = 2.53) to be later reported to child protection as alleged perpetrators for maltreatment of their own children (G2: 2nd generation), but only self-reported more neglect (AOR = 1.83). A similar discrepancy was found between self-report and official reports when their grown children (G2) were interviewed. Second generation children whose parents had child welfare histories had higher rates of official reports than those that did not, but there was no similar difference in self-report by group. The authors suggest that this is evidence of intergenerational SB, positing that the first generation’s involvement with child welfare resulted in surveillance that continued into adulthood as they became parents. The proposed mechanism of the surveillance is somewhat unclear, as child protection does not generate reports to itself.

### 1.2. Testing for Surveillance Bias: Chaffin & Bard’s Study

An in-depth attempt to directly test the existence and magnitude of SB was made by Chaffin and Bard [5] in a single published paper including two distinct evaluations. In the first, participants in three different programs (family support, parenting, and family preservation/reunification, *n* = 2618) were followed for three years. All official reports (not including the initial report which involved the families with CPS in the first place) were tracked and unique reports to CPS made by interveners working for these programs (“direct surveillance”) were noted. They estimated a total rate of unique surveillance bias of 1.4% (27% of children reported when intervener-generated reports were included vs. 26% reported when intervener-generated reports were excluded). If calculated as an increase among those cases with re-reports (only), the SB effect is about six percent. They reported a somewhat higher SB effect during the first few weeks of treatment, but this difference eroded quickly over time.

Their second analysis focused on a statewide nurse home visiting program based on the Olds Model. This second design, unlike the first, allowed comparison between the treatment sample (*n* = 9514) and a weighted, matched comparison group (*n* = 9507). Evidence for SB was found, but it again accounted for a maximum of 6% of official child abuse and neglect reports made within the treatment group. Chaffin and Bard conclude that “findings from both studies suggest that surveillance bias and different ways of handling surveillance reports in survival analyses mattered little in terms of overall findings.” [5] and that “…invoking surveillance as an explanation for disappointing findings seems questionable.”

### 1.3. The Present Study

The use of administrative data, especially those linked across agencies, is a useful and increasingly common approach to answer child maltreatment policy and practice questions [14]. This study uses two administrative data sources to test both the standard and intergenerational surveillance bias hypotheses. The two datasets include the “national data”, National Child Abuse and Neglect Data System (NCANDS) 2004–2015 Child File data, and more granular “regional” data from a longitudinal linked administrative data study done in a major Midwestern metropolitan area from 1993 through 2009.

## 2. Materials and Methods

The core assertion underlying SB is that SB reports are made by service providers, therefore this study focuses on proportions of reporter types in re-reports. Our hypotheses are based on the axiomatic assumption that if SB effects exist, they necessarily manifest in inflated proportions of professional reporters, particularly professional reporters who are mental health or social service (MHSS) providers in second reports to CPS. If SB is operative, a high proportion of professionals (And specifically, MHSS professionals) must obtain among re-reports—Reports made after SB has become operative due to child protection involvement. For example, if SB effects increase total re-reports by 50%, then an absolute minimum of 1/3 of all re-reports must be from MHSS sources. If substantial intergenerational SB exists, a much higher rate of professional (And MHSS) reporters must manifest among alleged perpetrators who themselves were officially reported as children.

### 2.1. Within-Generation Hypotheses

**Hypothesis 1** (44 state, 35 state and regional data)**.** Compared to first (Index) reports, re-reports will show a much higher proportion of unique reports from both (Uniquely) professional and (Uniquely) MHSS sources.

**Hypothesis 2** (35 state and regional data)**.** Compared to children not receiving services, children who receive services will show much higher proportions of re-reports from (Uniquely) MHSS and (Uniquely) professional sources.

**Hypothesis 3** (Regional data)**.** Using regional data, hypothesis 1 effects will be progressively stronger for children who received progressively more intensive services after their index report.

### 2.2. Intergenerational Hypothesis

**Hypothesis 4** (Regional data)**.** Among subjects who became parents, those previously reported as victims of maltreatment will have higher proportions of reports from professional reporters.

**Hypothesis 5** (Regional data)**.** Among subjects who became parents with prior histories of maltreatment reports, those who received child welfare services following a report will have higher proportions of reports from professional reporters compared to those with reports but never served.

### 2.3. Samples

NCANDS Child File data include one de-identified record for each report made, meaning multiple records can exist for an individual child over time, linkable across years. These data are used to create two datasets, the “44 state” (*n* = 825,763), and the “35 state” sample (*n* = 667,634). The 44 state sample excludes states with missing or incomplete report coverage during the study timeframe. The 35 state sample further excludes states that lacked usable child welfare service provision data. Children were selected into the sample if they were estimated to have been born in 2005 and had a first CPS report in their first seven years of life. Children who entered foster care as a function of their first report (Less than six percent of the sample) were excluded. Children were followed for three years after their index (First) report and all re-reports within that timeframe were noted. This requires linking multiple child file years which is possible due to a common unique identifier within each state.

The “regional” study included three groups of children born 1982–1994 (*n* = 12,409) created by linking official maltreatment report data with government income assistance data: (1) children living in families receiving income assistance with index reports prior to age 12 during the years 1993–1994, (2) children living in families receiving income assistance with without maltreatment reports matched to the first group by age and region, and (3) children with index reports prior to age 12 as of 1993–1994 who did not live in families receiving income assistance. In addition to child protection and income assistance data subjects were followed through 2009 by linking electronic data from several other agencies including education, health and mental health care billing, juvenile and criminal justice, runaway shelters and vital statistics. For the purpose of the present analyses, within generation analyses are limited to groups 1 and 3 who were age 16 or over as of January 2007. The age and year limitation was needed to capture “lifetime” risk of re-report by report source since reporter types were unavailable after 2007. Children who entered foster care but never exited were also excluded, leaving 7185 subjects for analyses of re-reports. For intergenerational analyses, female subjects who became parents were identified through linkage to vital statistics records (“G1”) and then to CPS reports involving themselves as alleged perpetrators of maltreatment of their children (“G2”). Female subjects were sampled from groups 1 and 2 so as to compare those with and without histories of maltreatment reports (*n* = 781).

Please see the attached Supplementary Materials for a more detailed description of these datasets, their derivation and management.

### 2.4. Within-Generation Variables

The outcome for within generation analyses for both data sources was the number and proportions of re-reports by reporter type. Detailed reporter types were combined into “MHSS” (“Mental Health” and “Social Services”) and also a broader “Professional” category, which included MHSS reporters as well as other specific kinds of mandated reporters, such as medical, law enforcement and education professionals. Remaining cases (“Non Professional” or “Unclassified”) were coded as “Other” (See Supplementary Materials). After Chaffin and Bard [5] re-reports by reporter type were further categorized as unique if all re-reports were only from MHSS sources (“Unique MHSS”) or all re-reports were only from professional sources (“Unique professional”). With regard to service delivery (35 state sample only) cases noted in NCANDS as receiving “Post Investigation Services” (POSTSERV = 1) were coded as “Served” with all other cases coded as “unserved”. The “regional” study data (*n* = 7185) allowed for a more exact categorization of intensity of services provided by CPS over time. CPS response was coded according to the most intensive level of intervention received after the index report: unsubstantiated and never served, substantiated and never served; family centered services (Case management); family preservation services (More intensive services); and entered but returned home from foster care.

### 2.5. Intergenerational Variables

Because it was possible for a group one G1 to have a later report after baseline, the child welfare service variable was created based on CPS records rather than baseline group status. The small sample size required collapsing those who received any in-home services with those who entered foster care (Served group). Because of the young age of some of the parents, it was possible for a G1 subject to become a parent while still receiving child welfare services themselves. Therefore the served group was further divided according to those who were not still eligible for child welfare services themselves and those who were. The outcome was any report involving a G2 child of G1. The multi-agency nature of the regional dataset allowed for the creation of a variable that indicated non-CPS system involvement between the time of pregnancy and the time of report as an alleged perpetrator. This was done limited to system involvement limited to G1 behaviors or conditions that might raise concerns about parenting (e.g., mental illness, criminal behavior or medical treatment for domestic violence or sexually transmitted infection (STI)). Additionally, receipt of income assistance during the same time period was examined as another potential point of contact with a mandated reporter but not a signal of parenting risk per se.

For the 44 state and 35 state data, a simple cumulative hazard approach was employed to examine re-reporting over time by type of re-report (MHSS, Unique MHSS, Professional and Unique Professional).

Within generation analyses of “regional” data included bivariate tests of report characteristics by prior childhood child protection involvement using chi-square or Fishers Exact according to sample size. Intergenerational analyses of first reports also included bivariate tests of significance for any report, reporter type, and possible surveillance by non-CPS systems.

## 3. Results

### 3.1. Within Generation Results

**Figure 1:**
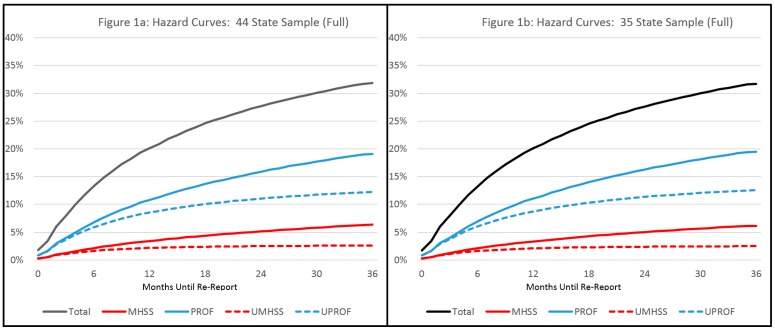
Hazard curves, percentage with re-report by re-reporter type: (**a**) full (44 State) sample; (**b**) 35 state sample. Note: “MHSS” indicates a mental health or social services reporter, “PROF” indicates any professional reporter (including MHSS), the prefix “U” indicates that the source was uniquely from that reporter type (e.g. “UMHSS” means reports were made only by a MHSS source or sources).

**Figure 2:**
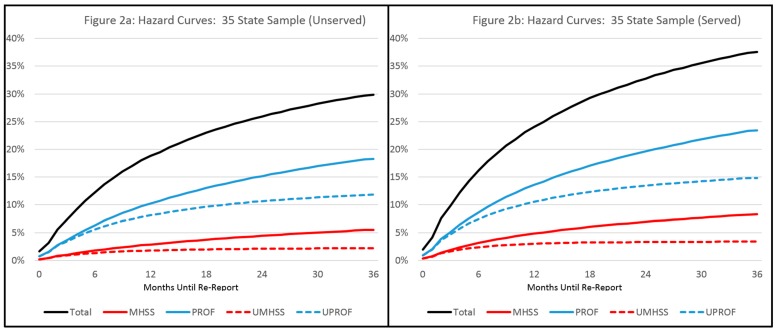
Hazard curves, percentage with re-report by re-reporter type: (**a**) Unserved cases; (**b**) Served cases. Note: “MHSS” indicates a mental health or social services reporter, “PROF” indicates any professional reporter (including MHSS), the prefix “U” indicates that the source was uniquely from that reporter type (e.g., “UMHSS” means reports were made only by a MHSS source or sources).

Hypotheses one through three were addressed using both the national and regional samples. With regard to the national data, the 44 state sample included 825,763 individuals of whom 31.82% had at least one re-report over three years. In the 35 state sample, there were 505,191 unserved individuals and 162,443 served individuals. Among those served, 37.55% had at least one re-report, while among the unserved cases, 29.81% had at least one re-report (See Table 1). Hazard curves showing re-reports over time can be found in Figure 1 (Full 44 state and 35 state samples) and Figure 2 (Served and unserved cases from 35 state sample).

There was little variability among the 44 and full 35 state samples with regard to the proportion of index reports from MHSS sources (11.16% vs. 10.32% respectively), or professional sources (52.37% and 56.29%, respectively). There was also little variance in proportions of re-reporter types between samples at 36 months post-index. The total number of unique MHSS re-report sources ranged from 7.37% (35 state unserved sample) to 9.04% (35 state served sample). The total number of unique professional re-reports ranged from 38.42% (45 state sample) to 39.64%% (35 state served sample). As can be seen from the above numbers, the percentage of unique MHSS re-reports was always lower than the percentage of MHSS index reports. The same was true for professional reports. It is important to note the small proportion of unique MHSS re-reports, which demonstrate that large SB effects on the order claimed (a 50% increase in re-reports) are mathematically impossible. Even if all unique MHSS re-reports were due to SB, the total magnitude of the SB effect would be less than ten percent at 36 months post-index for all samples.

The data did support hypothesis 2 to a limited degree. Two trends suggesting a real but small SB effect were found. First, the 35 state sample served cases had higher a higher percentage of total re-reports when compared to unserved cases (37.55% to 29.81%, respectively). This difference, however, was not mainly due to increased numbers of unique MHSS and professional re-reports. Proportions of re-reports from professionals were virtually identical between served and unserved groups (39.64% vs. 39.61%, respectively). Cases in the 35 state served sample did experience more unique MHSS re-reports than the unserved 35 state sample (9.04% vs. 7.37%, respectively). This supports a small SB effect among MHSS re-reporters but not the broader class of all professional re-reporters, as the SB hypothesis would predict.

Post-hoc analyses were performed to more precisely quantify this potential SB effect. These analyses were performed using the national data to estimate how total observed re-reports would have lessened if unique MHSS re-reports in the “served” subsample had increased only at the lesser rate seen among other reporter types. This was done mathematically by reducing the number of unique mental health social services reporters (UMHSS) re-reports among served cases to the lesser rate of re-reports among UMHSS unserved cases (See Supplementary Materials detailed procedure and calculations).

Using this procedure, we estimate that at 3 months post-index report, SB could plausibly increase re-reports among cases served at index by +4.54%. This should be interpreted to mean that if there would have been 100 re-reports (At three months) among served index cases without SB being present, SB could inflate the number of re-reports to 104.54 re-reports.

If all index cases are considered (Not just served cases) at three months post-index report, then the increase in re-reports reduces to +1.33%. This should be interpreted to mean that if there would have been 100 re-reports (at three months) among all index cases without SB being present, SB could inflate the number of re-reports to 101.33 re-reports.

These effects degrade rapidly over time. When the same analyses are made at 36 months, the corresponding increases due to SB are only 1.84% among served index cases and +0.52% among all index cases.

Moving away from the national data, the regional study provided more specific service and family data (See Table 2). Data in Table 2 are broken down by service type and poverty status of the family at baseline, to allow separate consideration of SB in poor and non-poor families. Among the 7185 index reports, 17.01% were from MHSS sources, with little variability by poverty status. Among re-reports, about twice as many re-reported cases had a re-report from a MHSS source (38.35%), but less than half as many had unique MHSS re-reports (only 7.66% of all re-reports). Surprisingly, this number was somewhat higher for non-poor children (10.38%) than for poor children (6.96%). With regard to hypothesis 1, we see that the percentage of UMHSS re-reports is lower than the percentage of MHSS index reports, so hypothesis 1 is not supported. Given that unique MHSS re-reports only constitute about one in fourteen re-reports, it is impossible for these re-reports to be causing a large increase in the total number of re-reports.

The regional data do provide some support for hypothesis 2, as CPS cases served by FCS or FPS (But not foster care) showed rates of UMHSS re-reports roughly twice as high (10.78% to 22.22%) as cases receiving no services (5.96% to 10.84%). This could be more evidence of a small SB effect. Contrary to expectation, foster care cases showed relatively low rates of unique MHSS re-reports.

Hypothesis 3 asserts that as service intensity increases, the proportion of unique MHSS re-reports will also increase. This hypothesis was not supported. Proportions of unique MHSS re-reports in less intensively served (FCS) and more intensively served (FPS) cases were quite similar , being, respectively, 19.47% vs. 22.22% for non-poor cases and 11.01% vs. 10.78% for served cases Foster care cases always had a relatively low proportion of unique MHSS re-reports, never exceeding 8%.

### 3.2. Intergenerational Results

Intergenerational issues could only be assessed using the regional sample (See Table 3). Column 1 describes the G1s’ childhood level of child protective service involvement: Never reported (28%), reported but never provided services (27.5%), reported and provided services but were not still eligible for child welfare services when they gave birth (25.2%), reported and provided services and were still eligible for child welfare or were in foster care when they gave birth (19.2%). G1s with prior histories of services following a report did have a higher rate of re-reports for maltreating G2s than those without CPS histories (See column 3). At first glance, this might appear supportive of the idea of intergenerational SB. However, were this increase due to SB, we would expect greater proportions of professional reporters among more intensively served children. This was not the case.

Hypothesis four stated that, among subjects who became parents, those previously reported as victims of maltreatment will have higher proportions of reports from professional reporters. Hypothesis five then posited that as service intensity increases, the proportion of professional reporters would increase. There were no statistically significant differences in proportions of reports by professional reporters by prior CPS history and, in fact, the trend was in the opposite direction (See column 4). Those G1s with no prior CPS history had a higher proportion of reports made by mandated reporters (Over 57%) than any other category (Between 32% and 53%). There was a trend for increased professional reporters among those G1s who had services (53.1% and 42.8%) following a CPS report compared to those with reports but not served (32.3%) but this was not statistically significant.

Childhood CPS involvement was indeed posited as a reason for a type of intergenerational surveillance bias by Widom and colleagues [7]. However, when looking at intergenerational surveillance, it is also possible that risky or concerning G1 behaviors or situations might place G1s in contact with professional providers separate from any child protection history. If this were to occur during pregnancy or following childbirth this could trigger concern among a professional reporter about the G2 child without the reporter having any knowledge of prior G1 maltreatment reports. A post-hoc assessment of potential surveillance by professional reporters subsequent to onset of pregnancy (And before the alleged report of G1 as perpetrator) was therefore examined by looking at service sector use related to issues that could place the G2 at risk: criminal behavior, mental illness or health care associated with domestic violence or STI. Over 35% of the G1s that were reported as alleged perpetrators were involved in systems for at least one of these issues. There was no statistically significant difference between those without CPS histories and those with CPS histories (See column 5). Although poverty is not a reason for reporting maltreatment, receipt of income assistance does mean that the G1s are in contact with another system with mandated reporters. When we added receipt of income assistance during the same time period (Not shown) the majority of all G1s were already being served by a non-CPS system prior to being reported as an alleged perpetrator, (68.7% to 90.5%) further diluting any possible “indirect SB” effect attributed to childhood CPS contact.

## 4. Discussion

Our findings show that large SB effects do not and cannot exist in the general CPS population. Axiomatically, for SB effects of +50% to obtain (As has been suggested in the literature) unique re-reports from MHSS providers would have to constitute at least one third of all re-reports (The “extra” 50%). This figure was not approached in our data. Even broadening the definition of SB to include all professionals does not help. All re-reports uniquely from any professional source never constitute more than 40% of all re-reports. This would require virtually every professional re-report, including those from law enforcement and education, to be cases of SB. This is clearly implausible. The higher overall rate of re-reports among served cases is not a new finding and is probably due to services being provided to more severely at-risk cases.

The percentage of unique re-reports from MHSS and professional sources was almost always lower than their proportion among index reports, not higher as SB would suggest. In the national data and the regional data, Hypothesis 2 was supported, but at a low magnitude. There were more unique MHSS re-reports among served cases, and this finding is consistent with a limited SB effect. The detected effect, however, only represents an increase in re-reporting of about two percent at three years, too small to be practically important in intervention studies, few of which are adequately powered to even detect such a difference. Our findings align closely with Chaffin & Bard [5] both in magnitude and in observing the same initial SB “bump”, in our case, at three months post-index report, which then deteriorates rapidly.

With regard to Hypotheses 2 and 3, that served cases, particularly more intensively served cases, will have higher proportions of re-reports from unique MHSS sources, the regional data also found limited evidence of a small SB effect, with cases served by FCS or FPS having somewhat higher proportions of re-reports from unique MHSS sources. Consistent with literature on a history of maltreatment being associated with greater risk of intergenerational maltreatment [15], G2s with parents with CPS histories had higher rates of reports as alleged perpetrators. Hypotheses 4 and 5, however, were also not supported. There was no evidence for SB causing reports through increasing the proportion of professional re-reporters based on the G1 childhood CPS history. Indeed, the majority of G1s were involved with at least one non-CPS system that would have provided more proximal potential for SB, likely washing out any indirect SB due to CPS contact.

### 4.1. Limitations

While we can estimate total SB effects among all CPS reports and among served CPS cases, we cannot parse out cases served specifically by home visitation. However, there is no empirical reason to believe that large SB effects exist within this subpopulation, and substantial prior evidence suggesting the opposite [5,6,13].

Another clear limitation has to do with our inability to track SB among first (Index) CPS reports. Some of the SB literature focuses on SB as it impacts re-reports. Some of the literature, however, focuses on SB effects on first (Index) reports. This is true of home visiting models which begin implementation prior to birth. Our study is directly generalizable only to effects on re-reports. However, our data also highlight a key point–unless any set of reports is radically different from the norm, and has vastly higher proportions of MHSS reports than we found, SB remains an untenable explanation for substantially inflated reports at any level.

Given the nature of the NCANDS system for identifying reporters, there were many (About one in five) “unclassified” reporters. “Unclassified includes anonymous, “other,” and unknown report sources.” [16] and were grouped with nonprofessional reports for purposes of the present analysis. It is theoretically possible that some professional report sources might not identify themselves or make anonymous reports and therefore be listed as “unknown”. Such reporters would be misclassified in this study as nonprofessionals and that could introduce error into our analyses. We were not able to examine SB for children who have a first report to CPS after age 11. This is not a serious limitation as virtually all prior studies purporting to show an SB effect have used populations of younger children. In the intergenerational data, both the G1 parents (Age of 17 and 27) and G2 children (Most under the age of 4) were still young. Thus G1 parents were still at risk of being reported as maltreating, and their “lifetime” report totals were continuing to rise. On the other hand, it may be reasonable to believe that intergenerational effects would be strongest while the parents are still younger, causing our timeframe to provide the most generous design for testing intergenerational SB.

Some readers may question our emphasis on MHSS reports. This was done for two reasons. First, all SB theory suggests that SB would reside among these reporters. Second, those small effects which manifested in our data did reside, as theory would predict, among MHSS reports, not among the broader category of professional reports. Our data clearly show that there is even less evidence for SB among the broader population of professional reporters than among the subset of MHSS reporters.

Another limitation always present in NCANDS data is failure to identify children who move across state boundaries. In such cases, when a previously reported child moves and is re-reported, an apparently new child is created in the new state. This is an unavoidable problem in NCANDS and will result in a small underestimation of re-reports [17]. Our limited timeframe minimizes this problem to some degree. Children who move may be less vulnerable to SB. As prior contacts are lost, SB becomes less likely. For this reason, any state boundary effects are likely to blind the study to cases with less likelihood of SB, meaning the obtained estimates may be very marginally skewed in favor of detecting SB.

Finally, questions may be raised about the representativeness of the 35 state sample. The additional states omitted in the smaller sample represent a broad range of regions and state types (See Supplementary Materials) and the hazard curves for the full 35 state sample closely mirror those of the 44 state sample (See Figure 1), providing some confidence that the smaller sample is not radically unrepresentative.

### 4.2. A Tempest in a Teapot

We find evidence that SB does exist, but at a trivial level. This is consistent with findings of Chaffin and Bard [5]. In summary, while SB may be interesting academically, and does, in fact appear to exist in a very small and temporary way, it is incapable of producing large effects on CPS re-reports in general. Concerns that CPS agency reports are unreliable outcome measures due to SB are not empirically supported at this time. Differences found between intergenerational self-report and official reports may have several alternative explanations including difficulties with retrospective recall [7] or increased risk of behaviors and conditions (e.g., criminality or mental illness) related to childhood maltreatment that are associated with impaired parenting [15,18,19,20,21] that may trigger second generation maltreatment reports that have nothing to do with lasting effects of child protection surveillance. Similar to the conclusions of Chaffin and Bard [5] we find little support for SB as an explanation or excuse for intervention study findings showing no measurable changes in official child abuse reporting rates. One possible exception would be in the case of intervention studies with very large samples and very high statistical power. In such cases, small magnitude SB effects might be observable. In any case, if SB is considered a potential confounding factor, it is incumbent upon the researcher to check and compare the composition of re-reporter types in treatment and control samples to determine if SB could plausibly be operative.

## 5. Conclusions

Despite widespread acceptance of surveillance bias as an important factor in CPS reporting, we find evidence of only a small effect which diminishes further over time. Our results are consistent across national and more granular regional data. At this point, the promulgation of surveillance bias as a meaningful dynamic in child welfare reporting is counter-empirical and unwarranted.

## Figures and Tables

**Table 1:** Types of index reporter and re-reporters over time (National data).

**Index Reports**		**Index Reports from MHSS Sources**	**Index Reports from Professional Sources**
44 State Sample (*n* = 825,763)		11.16%	52.37%
35 State Sample (*n* = 667,634)		10.52%	53.62%
35 State Sample (Served, *n* = 162,443)		11.13%	56.29%
35 State Sample (Unserved, *n* = 505,191)		10.32%	52.76%
**Re-reports**	**Percent of children with any re-report**	**Percent of all children with re-reports who have MHSS re-reports (Any/Unique)**	**Percent of all children with re-reports who have Professional re-reports (Any/Unique)**
44 State Sample			
Re-reports at 3 Months	7.94%	15.90%/13.94%	49.58%/46.20%
Re-reports at 12 Months	20.14%	17.04%/10.77%	53.61%/42.34%
Re-reports at 24 Months	27.66%	18.62%/9.04%	57.40%/39.94%
Re-reports at 36 Months	31.82%	19.97%/8.22%	59.97%/38.42%
35 State Sample			
Re-reports at 3 Months	7.93%	15.56%/13.57%	50.53%/47.10%
Re-reports at 12 Months	20.09%	16.66%/10.37%	54.99%/44.43%
Re-reports at 24 Months	27.59%	18.21%/8.66%	58.92%/41.08%
Re-reports at 36 Months	31.69%	19.49%/7.85%	61.55%/39.58%
35 State Sample, Served Children Only			
Re-reports at 3 Months	9.86%	18.94%/16.22%	51.73%/58.47%
Re-reports at 12 Months	24.07%	20.12%/12.51%	56.47%/43.73%
Re-reports at 24 Months	32.77%	21.12%/10.06%	59.89%/41.07%
Re-reports at 36 Months	37.55%	22.12%/9.04%	62.36%/39.64%
35 State Sample, Unserved Children Only			
Re-reports at 3 Months	7.31%	14.09%/12.42%	50.01%/46.89%
Re-reports at 12 Months	18.81%	15.24%/9.49%	54.38%/43.30%
Re-reports at 24 Months	25.93%	17.02%/8.10%	58.53%/41.09%
Re-reports at 36 Months	29.81%	18.42%/7.37%	61.23%/39.61%

**Table 2:** CW service level at index by reporter type and re-reports by reporter type according to baseline poverty (Regional Data).

	Index Cases			Children with Re-Reports					
Index Case CW Service Level	A	B	C	D	E	F	G	H	I
	*n*	Reporter Professional *n* (%)	Reporter MHSS *n* (%)	Total Re-Reported *n* (%)	Any Professional Re-Reports *n* (%)	Unique Professional Re-Reports *n* (%)	Any MHSS Re-Reports *n* (%)	Unique MHSS Re-Reports *n* (%)	Unique MHSS Re-Reports during CPS Service: *n* (%)
All Children	7185	2921 (40.65%)	1222 (17.01%)	4336	3010 (69.14%)	1065 (24.56%)	1663 (38.35%)	332 (7.66%)	11 (0.25%)
Nonpoor at Baseline (total)	2214	934 (49.12%)	357 (16.12%)	886	574 (64.79%)	276 (31.15%)	289 (32.62%)	92 (10.38%)	4 (0.45%)
Unsub/no srvs	1608	566 (35.20%)	209 (9.44%)	621 (38.62%)	381 (61.35%) ^1^	177 (28.50%) ^1^	189 (30.43%) ^1^	53 (8.53%) ^1^	n/a
Subst/no srvs	299	186 (62.21%)	72 (24.08%)	83 (27.76%)	63 (75.90%) ^1^	30 (36.14%)	31 (37.35%)	9 (10.84%)	n/a
FCS only	169	99 (58.58%)	41 (24.26%)	113 (66.86%)	76 (67.26%)	44 (38.94%) ^1^	43 (38.05%)	22 (19.47%) ^1,2^	4 (3.54%)
FPS	55	29 (52.73%)	9 (16.36%)	27 (49.09%)	22 (81.48%) ^1^	11 (40.74%)	14 (51.85%) ^1^	6 (22.22%) ^1,3^	0
Foster Care	83	54 (65.06%)	26 (31.33%)	42 (50.60%)	32 (76.19%)	14 (33.33%)	12 (28.57%)	<5% *^,2,3^	0
Poor at Baseline (total)	4971	1987 (39.97%)	865 (17.40%)	3450	2436 (70.61%)	789 (22.87%)	1374 (39.83%)	240 (6.96%)	7 (0.20%)
Unsub/no srvs	3308	991 (29.96%)	409 (12.36%)	2333 (70.53%)	1637 (70.17%) ^1^	489 (20.96%) ^1^	904 (38.75%) ^1^	139 (5.96%) ^1^	n/a
Subst/no srvs	679	442 (65.10%)	194 (28.57%)	413 (60.82%)	329 (79.66%) ^1,2^	104 (25.18%)	197 (47.70%) ^1,2^	31 (7.51%)	n/a
FCS only	481	242 (50.31%)	118 (24.53%)	345 (71.73%)	203 (58.84%) ^1–4^	94 (27.75%) ^1^	111 (32.17%) ^1–3^	38 (11.01%) ^1^	7 (2.03)%
FPS	226	122 (53.98%)	68 (30.09%)	167 (73.89%)	127 (76.05%) ^3^	54 (32.34%) ^1^	77 (46.11%) ^3,4^	18 (10.78%) ^1^	0
Foster Care	277	190 (68.59%)	76 (27.44%)	192 (69.31%)	140 (72.92%) ^4^	48 (25.00%)	85 (44.27%) ^4^	14 (7.29%)	0

Notes: “Index Case Service Type” provides non-overlapping counts indicating the most intensive level of CPS services received by the study subject (G1) as a child. Lower rows represent progressively more intensive services. Columns A–C refer to the Index report, Columns D–I refer to recurrence. Column I is the percent of Column D that were reported by unique MHSS while still actively receiving CW services. * exact number omitted for confidentiality cell size < 3. Significance tests for type of reporter among re-reports are chi-square or Fisher Exact when smaller cells. Cells with the same superscript number (i.e., 1–4) are significantly different from each other.

**Table 3:** Intergenerational report characteristics for adult female parents by level of their childhood CPS contact (Regional data).

1	2	3	4	5
G1 Child Protective Services Received Prior to Age 18	*n*	% G1 Reported to CPS for Allegedly Maltreating G2	% of G2 Reports (Column 3) Made by Professional Reporters	% of G2 Reports (Column 3) Made by MHSS Reporters
No CA/N report	219	9.59% ^1,2^	57.14%	9.52%
CA/N report with no services	215	14.42% ^3^	32.26%	9.68% ^1^
CA/N report with in-home or foster care services- Subjects who gave birth after 18th birthday and were not in foster care at when they gave birth.	197	16.24% ^2^	53.13%	31.43% ^1^
CA/N report with in-home or foster care services- subjects who gave birth prior to age 18 or were in foster care when they gave birth	150	23.33% ^1,3^	42.86%	21.88%

^1–3^ matching superscripts indicate groups are statistically significantly different according to chi-square or Fisher’s Exact *p* < 0.05. Notes: G1 refers to Generation **1** (parents), while G2 refers to reports made on their children. Column **1** describes childhood CPS involvement with the served groups in the last two rows divided by whether or not they were still minors when they gave birth and/or gave birth while still in foster care themselves. Column **2** provides number of female parents (G1s) according to any CPS services they received as a child. Column **3** provides the percent of G1 who were reported as alleged child abuse perpetrators of their children. Column **4** is the percent of G2 reports made by mandated reporters. Column **5** is the percent of G2 reports made by MHSS reporters.

## Supplementary Materials

The following are available online at www.mdpi.com/1660-4601/14/9/971/s1, Table S1: Cumulative re-reports by type, 44 state sample (*n* = 825,763); Table S2: Cumulative Re-Reports by Type, 35 State Sample (*n* = 667,634); Table S3: Cumulative Re-Reports by Type, 35 State Sample, Served Only (*n* = 162,433); Table S4: Cumulative Re-Reports by Type, 44 State Sample, Unserved Only (*n* = 505,191).
