# Supplementary material for: Surveillance Bias in Child Maltreatment: A Tempest in a Teapot
Source: Int J Environ Res Public Health. 2017 Aug 28;14(9):971. doi: 10.3390/ijerph14090971 (PMC5615508; doi:10.3390/ijerph14090971)
Supplement: Supplementary file 1 [file ijerph-14-00971-s001.pdf]

Supplemental Materials: Surveillance Bias in Child Maltreatment: A Tempest in a Teapot

These supplemental materials include more detailed descriptions of the national and regional samples used, and also present the monthly national re-report data.

1. National Data Description

The national data were drawn from the 2004–2015 NCANDS child files, available at (http://www.acf.hhs.gov/cb/research-data-technology/reporting-systems/ncands). These yearly child files were combined into a single longitudinal database. This is possible due to the presence of a consistent child identifier in all the files. Detailed reporter types in NCANDS data were combined into "MHSS" ("Mental Health" and "Social Services" only) "Other Professional" (all other professional reporters), "Non-Professional" (all other specified reporters) and "Other/Unknown" ("Other" or "Unknown" or "Missing"). It should be noted that because we excluded those (relatively few) children who entered foster care, post investigation services refers to any form of case management, family support or family preservation services provided directly by child welfare. It is, unfortunately, not possible to separate out specific in-home service types, nor are dates or length of service available. Those very few child fatalities in the dataset (less than two thousand per year) also were not included because NCANDS does not provide identifiers in such cases.

We followed a single-year age cohort of children estimated to have been born in 2005. Dates of birth are not available in NCANDS, but child age is recorded in years at the time the report was made. In constructing our single-year age cohort, we therefore selected children with a first report at age "0" in 2005, or a first report at age "1" in 2006, through a first report of age"6" in 2011. This would include children during the first seven years of their life (0-6.99 years old). It was possible that children aged "0" in 2005 could have also had a report in 2004. The 2004 data were therefore checked and such children were excluded, in order to make sure our index reports were true first reports. In simple terms, the 2005-2011 files were consulted (and checked against the 2004 file) to create a list of all children estimated to have been born in 2005 who had a first child abuse and neglect report. The child's first report could be at any time from age 0 up until (but not including) their seventh birthday.

We then checked to see which children in this sample had re-reports within three years, using reports from Federal years through 2014. We followed all children for exactly three years to avoid variable re-report timeframes based on the age at which the child received their first report. If we had simply tracked all subsequent reports, then young children would have been followed for much longer than older children.

One persistent problem in NCANDS data is that data from a given federal year (say, 2014) is not completely present in that year's (2014) submission. This is because open cases or unprocessed cases are often delayed. Such cases are commonly sent the following year. For this reason, we used the 2015 children's data file to supplement our data to the extent that it included "delayed" cases which were actually reported in Federal 2014.

In summary, we included all children estimated to have been born in 2005 who had a first report of maltreatment prior to their seventh birthday. We then followed each child for three years after that initial report and recorded any re-reports.

NCANDS state inclusion/exclusion: The NCANDS Child Files were not routinely provided prior to the past fifteen years, and many states have "gaps" even during that timeframe. Since 2004, only 44 states (including the District of Columbia as a "state") have provided continuous non-missing data.

The problem was compounded when service data was considered. Unfortunately, NCANDS service receipt data have been even more unevenly collected across states. Only 35 (not including the District of Columbia) of our 44 states have existing service ("POSTSERV" variable) data which are sufficiently stable over time (no missing years, no suspiciously large adjacent year variation) to allow for inclusion in the service-specific analyses.

We therefore use two distinct samples, a "44 state sample" (including DC as a "state") for analyses simply looking at professional reporters without controlling for services and a reduced "35 State sample" for analyses comparing served and not served cases. Starting with all fifty states, Puerto Rico and DC, we excluded the following states from the 44 state sample: AK, AL, MD, MI, ND, OR, PR, and WI. The 35 state sample was further reduced by omitting DC, GA, ID, IN, MD, NC, NY, PA and SD.

2. Regional Data Description

These data are drawn from a larger longitudinal study using data from the St. Louis metropolitan area beginning in the early 1990's and funded by the National Institute of Mental Health (R0MH 06 1733-04 A1). This study used child protective services reports and income maintenance (AFDC and TANF) records to select all children aged 0-11 (inclusive) with a first child maltreatment report during 1993 or 1994, as well as a matched comparison group receiving public assistance but with with no maltreatment reports during that period. Children were followed through midyear, 2006. The data held by the State of Missouri were already linked, because the CPS and Income Maintenance files share a common child identifier. Children who were reported for fatality or who died within the first week were excluded from the study. To allow for independence of observations, one child per family was randomly selected ($N = 12{,}409$) when multiple children were present. Exclusion critieria (see main article) further reduced the sample to 7,185. For intergenerational analyses, only 781 families met inclusion criteria (see main article). For more information on this dataset, see Jonson-Reid, Melissa, Brett Drake, and Patricia L. Kohl. "Is the overrepresentation of the poor in child welfare caseloads due to bias or need?" *Children and Youth Services Review* 31.3 (2009): 422-427.

3. Monthly Data-Supplementary Tables

Three tables have been appended showing the cumulative reports at the national level, using the 44 state sample (Table S1) the full 35 state sample (Table S2) and the served and unserved cases within the 35 state sample (Tables S3 and S4). Each includes cumulative monthly counts for any report, reports from MHSS sources, reports from Non-MHSS sources, reports from Professional and Non-Professional sources.

4. Post-Hoc Analysis: Estimating the magnitude of SB

For the 36 month samples, the following procedure was used, with the 35 state sample as the data source. This analysis is built on the following premises (using 36 month figures for the next four numeric points):

1) Served UMHSS cases had 1.545 times as many rereports as unserved cases.
2) Remaining (non-UMHSS) cases had "only" 1.237 times as many rereports as unserved cases.
3) This difference (1.545 vs. 1.237) could plausibly be due to SB among UMHSS cases.
4) We therefore adjust down the total number of re-reports among UMHSS cases by using the lower rate (1.237) to model what might have happened in the absence of SB. The difference between totals is the number of "excess" reports plausibly due to SB.

We repeat this calculation process below for the 3 month data.

5. Procedure (36 month re-reporting)

The rate of unique reports from MHSS sources was calculated (UMHSS). The number of all other reports was calculated (Other). This was done for both served and unserved samples.

	Unserved 35 State Sample Rate	Served 35 State Sample Rate	Increase
**Other**	0.276	0.342	+23.7035942%
**UMHSS**	0.022	0.034	+54.5033917%

We can therefore make the following calculations:

1. While served cases always had higher re-report rates, this increase was more pronounced among UMHSS cases (+54.5033917%) than among other cases (+23.7035942%).
2. If served UMHSS cases had only increased at the lesser rate (the presumably "non-SB influenced" rate of 1.237035942) instead of the observed rate (1.545033917) then their rate of re-reporting would have been reduced to 1.237035942/1.545033917 or 80.0652936% of the actual observed re-reports among UMHSS cases.
3. Given that there were 5515 unique MHSS served re-reports (see Table S-3), this adjustment would reduce those re-reports to 5515*.800652936 or 4416 adjusted "non-SB influenced" total re-reports, a reduction of 1099 reports.
4. There were 211,582 total re-reports made in the 35 state sample (see Table S-2). The estimated revised "non-SB influenced" re-report total is 211,582−1099 = 210,483.
5. For purposes of understanding *SB effects upon rereports across the population of all index reports received by CPS* (both served and unserved), the "SB-influenced" rate divided by the "non-SB influenced" rate is (211,582/210,483) or 100.52%, suggesting that SB influence may plausibly account for an increase of 0.52% among all index reports (served and unserved cases).
6. For purposes of understanding *re-reports among only those cases previously served by CPS* (served cases only – unserved cases are omitted), the "SB-influenced" rate is (60,997/59,898) or 101.84%, suggesting that SB influence may plausibly account for an increase of 1.84% among that subset of index reports served by CPS.

6. Procedure (3 month re-reporting)

Again, the rate of unique reports from MHSS sources was calculated (UMHSS). The number of all other reports was calculated (Other). This was done for *both* served and unserved samples.

	Unserved 35 State Sample Rate	Served 35 State Sample Rate	Rate of Increase
**Other**	0.064	0.083	1.291663174
**UMHSS**	0.009	0.016	1.763644408

We can therefore make the following calculations:

1. While served cases always had higher re-report rates, this increase was more pronounced among UMHSS cases (+76.3644408%) than among other cases (+29.1663174%).
2. If served UMHSS cases had only increased at the lesser rate (the presumably "non-SB influenced" rate of 1.291663174) instead of the observed rate (1.763644408) then their rate of re-reporting would have been reduced to 1.291663174/1.763644408 or 0.732382995% of the actual observed re-reports among UMHSS cases.
3. Given that there were 2599 unique MHSS served re-reports (see Table S-3), this adjustment would reduce those re-reports to 2599*0.732382995 or 1903 adjusted "non-SB influenced" total re-reports, a reduction of 696 reports.

Table S1. Cumulative re-reports by type, 44 state sample (*n* = 825,763).

Month	Any Re-report		MHSS Re-reports		Professional Re-reports		Unique MHSS Re-reports		Unique Professional Re-reports	
	#	%	#	%	#	%	#	%	#	%
0	14510	1.76%	2160	0.26%	6892	0.83%	2126	0.26%	6841	0.83%
1	27931	3.38%	4389	0.53%	13513	1.64%	4132	0.50%	13100	1.59%
2	50139	6.07%	7960	0.96%	24696	2.99%	7212	0.87%	23453	2.84%
3	65551	7.94%	10424	1.26%	32502	3.94%	9135	1.11%	30287	3.67%
4	81591	9.88%	13060	1.58%	40844	4.95%	10947	1.33%	37190	4.50%
5	96643	11.70%	15539	1.88%	48851	5.92%	12467	1.51%	43513	5.27%
6	109458	13.26%	17723	2.15%	55814	6.76%	13624	1.65%	48693	5.90%
7	121573	14.72%	19902	2.41%	62690	7.59%	14733	1.78%	53582	6.49%
8	131658	15.94%	21737	2.63%	68417	8.29%	15557	1.88%	57443	6.96%
9	141948	17.19%	23647	2.86%	74411	9.01%	16362	1.98%	61350	7.43%
10	150213	18.19%	25263	3.06%	79363	9.61%	16959	2.05%	64488	7.81%
11	159597	19.33%	27017	3.27%	85022	10.30%	17535	2.12%	67967	8.23%
12	166313	20.14%	28333	3.43%	89155	10.80%	17918	2.17%	70418	8.53%
13	172342	20.87%	29532	3.58%	93063	11.27%	18255	2.21%	72619	8.79%
14	179981	21.80%	31170	3.77%	97910	11.86%	18690	2.26%	75350	9.12%
15	185543	22.47%	32328	3.91%	101536	12.30%	18948	2.29%	77251	9.36%
16	191944	23.24%	33761	4.09%	105702	12.80%	19245	2.33%	79399	9.62%
17	197277	23.89%	34967	4.23%	109189	13.22%	19474	2.36%	81146	9.83%
18	203007	24.58%	36248	4.39%	112989	13.68%	19706	2.39%	83018	10.05%
19	207664	25.15%	37349	4.52%	116286	14.08%	19875	2.41%	84598	10.24%
20	212005	25.67%	38417	4.65%	119356	14.45%	20088	2.43%	86052	10.42%
21	216808	26.26%	39564	4.79%	122677	14.86%	20232	2.45%	87549	10.60%
22	220560	26.71%	40504	4.91%	125330	15.18%	20365	2.47%	88673	10.74%
23	224978	27.24%	41626	5.04%	128578	15.57%	20544	2.49%	90136	10.92%
24	228434	27.66%	42526	5.15%	131117	15.88%	20654	2.50%	91244	11.05%
25	232588	28.17%	43572	5.28%	134156	16.25%	20767	2.51%	92431	11.19%
26	235683	28.54%	44447	5.38%	136538	16.53%	20867	2.53%	93339	11.30%
27	239397	28.99%	45462	5.51%	139282	16.87%	20966	2.54%	94339	11.42%
28	242415	29.36%	46283	5.60%	141595	17.15%	21024	2.55%	95181	11.53%
29	245441	29.72%	47183	5.71%	143907	17.43%	21104	2.56%	96060	11.63%
30	248686	30.12%	48136	5.83%	146380	17.73%	21197	2.57%	96940	11.74%
31	251420	30.45%	48940	5.93%	148466	17.98%	21288	2.58%	97735	11.84%
32	254414	30.81%	49807	6.03%	150862	18.27%	21354	2.59%	98640	11.95%
33	256840	31.10%	50585	6.13%	152772	18.50%	21446	2.60%	99337	12.03%
34	259681	31.45%	51503	6.24%	155046	18.78%	21539	2.61%	100104	12.12%
35	261822	31.71%	52170	6.32%	156798	18.99%	21589	2.61%	100708	12.20%
36	262755	31.82%	52467	6.35%	157562	19.08%	21597	2.62%	100962	12.23%

"#"represents the cumulative number of children who have had a report of the indicated type up through the month in question. The "%" figure shows the cumulative percentage of children in the total sample (N=613,210) who have had such a report. Note: Multiple reports may occur each month (e.g. professional and non-professional).

1. There were 52934 total re-reports made (see Table S-2). The estimated revised "non-SB influenced" re-report total is 52934−696 = 52238.
2. For purposes of understanding *SB effects upon rereports across the population of all index reports received by CPS* (both served and unserved), the "SB-influenced" rate divided by the "non-SB influenced" rate is (52934/52238) or 101.33%, suggesting that SB influence may plausibly account for an increase of 0.52% among all index reports (served and unserved cases).

3. For purposes of understanding *re-reports among only those cases previously served by CPS* (served cases only – unserved cases are omitted), the "SB-influenced" rate is (16025/15329) or 104.54%, suggesting that SB influence may plausibly account for an increase of 4.54% among that subset of index reports served by CPS (served cases only).

Table S2. Cumulative re-reports by type, 35 state sample (n = 667,634).

Month	Any Re-report		MHSS Re-reports		Professional Re-reports		Unique MHSS Re-reports		Unique Professional Re-reports	
	#	%	#	%	#	%	#	%	#	%
0	11431	1.71%	1680	0.25%	5520	0.83%	1651	0.25%	5479	0.82%
1	22518	3.37%	3486	0.52%	11077	1.66%	3270	0.49%	10737	1.61%
2	40590	6.08%	6337	0.95%	20366	3.05%	5724	0.86%	19349	2.90%
3	52934	7.93%	8236	1.23%	26748	4.01%	7182	1.08%	24932	3.73%
4	65829	9.86%	10324	1.55%	33651	5.04%	8603	1.29%	30656	4.59%
5	77978	11.68%	12292	1.84%	40315	6.04%	9786	1.47%	35892	5.38%
6	88289	13.22%	14011	2.10%	46074	6.90%	10690	1.60%	40200	6.02%
7	98135	14.70%	15743	2.36%	51817	7.76%	11544	1.73%	44298	6.64%
8	106241	15.91%	17180	2.57%	56562	8.47%	12165	1.82%	47494	7.11%
9	114584	17.16%	18672	2.80%	61555	9.22%	12768	1.91%	50779	7.61%
10	121245	18.16%	19944	2.99%	65685	9.84%	13198	1.98%	53368	7.99%
11	128692	19.28%	21311	3.19%	70327	10.53%	13636	2.04%	56199	8.42%
12	134125	20.09%	22351	3.35%	73756	11.05%	13909	2.08%	58244	8.72%
13	139008	20.82%	23303	3.49%	77021	11.54%	14159	2.12%	60100	9.00%
14	145162	21.74%	24603	3.69%	81048	12.14%	14513	2.17%	62389	9.34%
15	149621	22.41%	25525	3.82%	84053	12.59%	14713	2.20%	63966	9.58%
16	154815	23.19%	26667	3.99%	87537	13.11%	14949	2.24%	65784	9.85%
17	159114	23.83%	27626	4.14%	90440	13.55%	15130	2.27%	67256	10.07%
18	163758	24.53%	28618	4.29%	93579	14.02%	15301	2.29%	68828	10.31%
19	167521	25.09%	29485	4.42%	96339	14.43%	15427	2.31%	70177	10.51%
20	170979	25.61%	30340	4.54%	98863	14.81%	15596	2.34%	71378	10.69%
21	174860	26.19%	31239	4.68%	101619	15.22%	15695	2.35%	72618	10.88%
22	177896	26.65%	31972	4.79%	103784	15.55%	15789	2.36%	73565	11.02%
23	181410	27.17%	32842	4.92%	106428	15.94%	15881	2.38%	74735	11.19%
24	184223	27.59%	33542	5.02%	108544	16.26%	15962	2.39%	75680	11.34%
25	187526	28.09%	34349	5.14%	111019	16.63%	16044	2.40%	76664	11.48%
26	189992	28.46%	35030	5.25%	112985	16.92%	16114	2.41%	77417	11.60%
27	192966	28.90%	35825	5.37%	115251	17.26%	16191	2.43%	78242	11.72%
28	195371	29.26%	36467	5.46%	117183	17.55%	16235	2.43%	78964	11.83%
29	197809	29.63%	37173	5.57%	119090	17.84%	16305	2.44%	79701	11.94%
30	200399	30.02%	37918	5.68%	121116	18.14%	16364	2.45%	80427	12.05%
31	202575	30.34%	38520	5.77%	122822	18.40%	16423	2.46%	81096	12.15%
32	205001	30.71%	39197	5.87%	124803	18.69%	16480	2.47%	81872	12.26%
33	206932	30.99%	39778	5.96%	126347	18.92%	16529	2.48%	82424	12.35%
34	209140	31.33%	40479	6.06%	128196	19.20%	16575	2.48%	83057	12.44%
35	210838	31.58%	40994	6.14%	129619	19.41%	16610	2.49%	83552	12.51%
36	211582	31.69%	41228	6.18%	130234	19.51%	16616	2.49%	83749	12.54%

"#" represents the cumulative number of children who have had a report of the indicated type up through the month in question. "%" shows the cumulative percentage of children in the total sample (n = 613,210) who have had such a report. Note: Multiple reports may occur each month (e.g. professional and non-professional).

Table S3. Cumulative re-reports by type, 35 state sample, served only (n = 162,433).

Month	Any Re-report		MHSS Re-reports		Professional Re-reports		Unique MHSS Re-reports		Unique Professional Re-reports	
	#	%	#	%	#	%	#	%	#	%
0	3199	1.97%	538	0.33%	1543	0.95%	530	0.33%	1530	0.94%
1	6680	4.11%	1220	0.75%	3329	2.05%	1138	0.70%	3226	1.99%
2	12301	7.57%	2323	1.43%	6313	3.89%	2070	1.27%	5949	3.66%
3	16025	9.86%	3035	1.87%	8290	5.10%	2599	1.60%	7625	4.69%
4	19863	12.23%	3815	2.35%	10433	6.42%	3136	1.93%	9370	5.77%
5	23338	14.37%	4514	2.78%	12383	7.62%	3555	2.19%	10861	6.69%
6	26234	16.15%	5121	3.15%	14074	8.66%	3876	2.39%	12104	7.45%
7	28979	17.84%	5687	3.50%	15722	9.68%	4148	2.55%	13229	8.14%
8	31275	19.25%	6162	3.79%	17095	10.52%	4353	2.68%	14114	8.69%
9	33603	20.69%	6617	4.07%	18523	11.40%	4499	2.77%	15030	9.25%
10	35461	21.83%	7069	4.35%	19707	12.13%	4651	2.86%	15726	9.68%
11	37496	23.08%	7518	4.63%	21023	12.94%	4789	2.95%	16472	10.14%
12	39098	24.07%	7868	4.84%	22080	13.59%	4890	3.01%	17098	10.53%
13	40496	24.93%	8163	5.03%	23022	14.17%	4958	3.05%	17640	10.86%
14	42281	26.03%	8556	5.27%	24192	14.89%	5052	3.11%	18284	11.26%
15	43517	26.79%	8861	5.45%	25076	15.44%	5104	3.14%	18718	11.52%
16	44953	27.67%	9202	5.66%	26048	16.04%	5158	3.18%	19204	11.82%
17	46203	28.44%	9499	5.85%	26876	16.54%	5211	3.21%	19629	12.08%
18	47521	29.25%	9804	6.04%	27770	17.10%	5249	3.23%	20070	12.36%
19	48575	29.90%	10041	6.18%	28513	17.55%	5265	3.24%	20428	12.58%
20	49497	30.47%	10307	6.34%	29197	17.97%	5305	3.27%	20718	12.75%
21	50574	31.13%	10587	6.52%	29958	18.44%	5324	3.28%	21027	12.94%
22	51408	31.65%	10800	6.65%	30540	18.80%	5329	3.28%	21287	13.10%
23	52422	32.27%	11053	6.80%	31279	19.26%	5353	3.30%	21603	13.30%
24	53232	32.77%	11241	6.92%	31879	19.62%	5355	3.30%	21860	13.46%
25	54191	33.36%	11472	7.06%	32562	20.05%	5363	3.30%	22105	13.61%
26	54887	33.79%	11666	7.18%	33110	20.38%	5378	3.31%	22315	13.74%
27	55734	34.31%	11895	7.32%	33748	20.78%	5394	3.32%	22545	13.88%
28	56377	34.71%	12090	7.44%	34288	21.11%	5401	3.32%	22708	13.98%
29	57050	35.12%	12306	7.58%	34841	21.45%	5428	3.34%	22930	14.12%
30	57809	35.59%	12498	7.69%	35408	21.80%	5439	3.35%	23145	14.25%
31	58424	35.97%	12690	7.81%	35889	22.09%	5464	3.36%	23343	14.37%
32	59087	36.37%	12884	7.93%	36449	22.44%	5476	3.37%	23558	14.50%
33	59633	36.71%	13077	8.05%	36898	22.71%	5495	3.38%	23714	14.60%
34	60266	37.10%	13267	8.17%	37419	23.04%	5500	3.39%	23895	14.71%
35	60780	37.42%	13424	8.26%	37851	23.30%	5514	3.39%	24039	14.80%
36	60997	37.55%	13495	8.31%	38037	23.42%	5515	3.40%	24099	14.84%

"#" represents the cumulative number of children who have had a report of the indicated type up through the month in question. "%" shows the cumulative percentage of children in the total sample (n = 613,210) who have had such a report. Note: Multiple reports may occur each month (e.g. professional and non-professional).

Table S4. Cumulative re-reports by type, 44 state sample, unserved only (n = 505,191).

Month	Any Re-report		MHSS Re-reports		Professional Re-reports		Unique MHSS Re-reports		Unique Professional Re-reports	
	#	%	#	%	#	%	#	%	#	%
0	8232	1.63%	1142	0.23%	3977	0.79%	1121	0.22%	3949	0.78%
1	15838	3.14%	2266	0.45%	7748	1.53%	2132	0.42%	7511	1.49%
2	28289	5.60%	4014	0.79%	14053	2.78%	3654	0.72%	13400	2.65%
3	36909	7.31%	5201	1.03%	18458	3.65%	4583	0.91%	17307	3.43%
4	45966	9.10%	6509	1.29%	23218	4.60%	5467	1.08%	21286	4.21%
5	54640	10.82%	7778	1.54%	27932	5.53%	6231	1.23%	25031	4.95%
6	62055	12.28%	8890	1.76%	32000	6.33%	6814	1.35%	28096	5.56%
7	69156	13.69%	10056	1.99%	36095	7.14%	7396	1.46%	31069	6.15%
8	74966	14.84%	11018	2.18%	39467	7.81%	7812	1.55%	33380	6.61%
9	80981	16.03%	12055	2.39%	43032	8.52%	8269	1.64%	35749	7.08%
10	85784	16.98%	12875	2.55%	45978	9.10%	8547	1.69%	37642	7.45%
11	91196	18.05%	13793	2.73%	49304	9.76%	8847	1.75%	39727	7.86%
12	95027	18.81%	14483	2.87%	51676	10.23%	9019	1.79%	41146	8.14%
13	98512	19.50%	15140	3.00%	53999	10.69%	9201	1.82%	42460	8.40%
14	102881	20.36%	16047	3.18%	56856	11.25%	9461	1.87%	44105	8.73%
15	106104	21.00%	16664	3.30%	58977	11.67%	9609	1.90%	45248	8.96%
16	109862	21.75%	17465	3.46%	61489	12.17%	9791	1.94%	46580	9.22%
17	112911	22.35%	18127	3.59%	63564	12.58%	9919	1.96%	47627	9.43%
18	116237	23.01%	18814	3.72%	65809	13.03%	10052	1.99%	48758	9.65%
19	118946	23.54%	19444	3.85%	67826	13.43%	10162	2.01%	49749	9.85%
20	121482	24.05%	20033	3.97%	69666	13.79%	10291	2.04%	50660	10.03%
21	124286	24.60%	20652	4.09%	71661	14.18%	10371	2.05%	51591	10.21%
22	126488	25.04%	21172	4.19%	73244	14.50%	10460	2.07%	52278	10.35%
23	128988	25.53%	21789	4.31%	75149	14.88%	10528	2.08%	53132	10.52%
24	130991	25.93%	22301	4.41%	76665	15.18%	10607	2.10%	53820	10.65%
25	133335	26.39%	22877	4.53%	78457	15.53%	10681	2.11%	54559	10.80%
26	135105	26.74%	23364	4.62%	79875	15.81%	10736	2.13%	55102	10.91%
27	137232	27.16%	23930	4.74%	81503	16.13%	10797	2.14%	55697	11.02%
28	138994	27.51%	24377	4.83%	82895	16.41%	10834	2.14%	56256	11.14%
29	140759	27.86%	24867	4.92%	84249	16.68%	10877	2.15%	56771	11.24%
30	142590	28.22%	25420	5.03%	85708	16.97%	10925	2.16%	57282	11.34%
31	144151	28.53%	25830	5.11%	86933	17.21%	10959	2.17%	57753	11.43%
32	145914	28.88%	26313	5.21%	88354	17.49%	11004	2.18%	58314	11.54%
33	147299	29.16%	26701	5.29%	89449	17.71%	11034	2.18%	58710	11.62%
34	148874	29.47%	27212	5.39%	90777	17.97%	11075	2.19%	59162	11.71%
35	150058	29.70%	27570	5.46%	91768	18.17%	11096	2.20%	59513	11.78%
36	150585	29.81%	27733	5.49%	92197	18.25%	11101	2.20%	59650	11.81%

"#" represents the cumulative number of children who have had a report of the indicated type up through the month in question. "%" shows the cumulative percentage of children in the total sample (n = 613,210) who have had such a report. Note: Multiple reports may occur each month (e.g. professional and non-professional).
